# Genetic analysis of platelet-derived growth factor receptor-like gene (*PDGFRL*) polymorphism and melanin traits in Lanping black-boned sheep (*Ovis aries*)

**DOI:** 10.5194/aab-67-383-2024

**Published:** 2024-08-06

**Authors:** Dan Yue, Chaochao Peng, Sameeullah Memon, Azeem Iqbal, Heli Xiong, Xiaoming He, Ying Lu, Weidong Deng

**Affiliations:** 1 Faculty of Animal Science and Technology, Yuxi Agriculture Vocation–Technical College, Yuxi, Yunnan 653106, P.R. China; 2 Yunnan Provincial Key Laboratory of Animal Nutrition and Feed, Faculty of Animal Science and Technology, Yunnan Agricultural University, Kunming 650201, P.R. China; 3 Yunnan Animal Science and Veterinary Institute, Kunming 650224, P.R. China

## Abstract

In the intricate tapestry of Yunnan Province's biodiversity, the Lanping black-boned sheep (LPBB) emerges as a captivating enigma, distinguished by its profound melanin pigmentation adorning both its skin and its internal organs. Initially cataloged in the 1950s within the confines of Lanping County, this exceptional mammalian species presents a scarcity and uniqueness that extends beyond its geographic origins. Here, we collected 100 blood samples from Lanping black-boned sheep along with 50 samples each from Lanping normal sheep (LPN) and Huize normal sheep (HZN), all sourced from Yunnan Province. Our investigation focused on the association between the platelet-derived growth factor receptor-like gene (*PDGFRL*) polymorphism and the distinctive melanin characteristics observed in Lanping black-boned sheep. Utilizing UV–visible spectrophotometry, we assessed the melanin indexes present, such as tyrosinase activity and true melanin in the sheep blood, and the results demonstrated a significant elevation in melanin indexes for Lanping black-boned sheep compared to the control group ( P<0.05 ). We also identified three synonymous mutation sites within a partial 1128 bp exon fragment of the gene-encoding *PDGFRL* (EX2-G408A, EX5-T184C, and EX5-G222T). Notably, Lanping black-boned sheep, harboring genotypes GG, TT, and GG at these specific sites, showcased a pronounced surge in tyrosinase activity, eumelanin / total melanin ratios, and plasma colorimetric values when contrasted with the control group ( P<0.05 ). The discernment of GG, TT, and GG as the prevailing genotypes at their respective genetic loci in Lanping black-boned sheep heralds a breakthrough in our understanding of the genetic markers associated with black pigmentation. However, all three loci are silent mutations and do not alter the phenotypic changes. Whether they affect changes in melanin content through other metabolic pathways requires further study. In conclusion, the *PDGFRL* gene was silenced by mutations in our study and affected blood melanin levels. However, the gene did not undergo a missense mutation that altered the phenotypic changes, and the exact channel through which the changes in melanin content were affected needs to be further verified.

## 1 Introduction

Lanping County, Yunnan Province, China, is located in the southernmost part of the Three Parallel Rivers natural World Heritage Site. The elevation of Lanping County ranges from 3688 m at the summit to 2237 m at the foot of the mountain, and the average annual temperature is 10.7 °C (Zhu et al., 2023). In the 1950s, a special species of sheep, the Lanping black-boned sheep (LPBB), was discovered here by chance. LPBB distinguishes itself through the distinctive black pigmentation exhibited on both skin and internal organs, which is a striking departure from the reddish hues observed in Lanping normal sheep (LPN) (Deng et al., 2008; Sun et al., 2020). Noteworthy is the parallel pigmentation pattern shared with the Chinese silky fowl, yet LPBB stands as the solitary mammalian specimen documented to harbor a substantial melanin reservoir within its body (Xiong et al., 2020). The hematological peculiarity of LPBB, characterized by dark black blood, further underscores its unique physiological attributes and sets it apart among the local sheep breeds in Lanping. Renowned for its delectably tender meat quality and the absence of any discernible mutton odor, LPBB has garnered distinction as a novel genetic resource sanctioned by the Chinese Ministry of Agriculture and Rural Affairs (Raza et al., 2022; Zhou et al., 2023; Gao et al., 2023; Wu et al., 2023). The official recognition and endorsement of LPBB by the National Animal and Poultry Genetic Resources Appraisal Committee in October 2009, culminating in its inclusion in prestigious lists such as the “National List of Animal and Poultry Genetic Resources”, “China Rare Animal Breeds List”, and “World Rare Animal Species List” accentuate its significance on a global scale (Fan et al., 2022). Despite its elevated status, research on LPBB remains notably sparse in the National Center for Biotechnology Information (NCBI) database, with only 17 identified articles (https://www.ncbi.nlm.nih.gov/search/all/?term=black-boned sheep, last access: 20 July 2023).

Notably, infrared spectroscopy (IR) graphs have revealed identical main melanin molecular structures in LPBB and silky fowls (Deng et al., 2009; Darwish et al., 2018). Additionally, LPBB melanin exhibits insolubility and other melanin characteristics (Deng et al., 2009). Previous studies have indicated that the presentation of *PDGFRL* decreases as melanoma progresses from non-metastatic to metastatic stages, suggesting that *PDGFRL* may play a role in restricting melanoma growth by inhibiting melanocyte metastasis (Jiang et al., 2022; Liu et al., 2022a; Mazzio et al., 2018; Xu et al., 2020a). In our previous investigations, we explored the genetic differentiation between LPBB and normal sheep through the evaluation of the fixation index (FST) (Deng et al., unpublished data). Interestingly, we observed higher values of FST in the region containing the *PDGFRL* gene. However, the specific impact of the *PDGFRL* gene on the amount of melanin in LPBB remains unknown. Hence, in this study, we set out to investigate the association between *PDGFRL* gene polymorphism and the amount of melanin in LPBB, with the ultimate goal of comprehending the underlying interaction and establishing a theoretical foundation for studying LPBB's germplasm origin and melanin traits.

## 2 Materials and methods

### 2.1 Ethical statement

This study was conducted in strict accordance with the guidelines outlined by the International Cooperation Committee of Animal Welfare (ICCAW) for the care and use of animals in China. All experiments were carried out in the Key Laboratory of Animal Nutrition and Feed Science of Yunnan Province.

### 2.2 Sample collection

In 2020, 100 blood samples (of 50 males and 50 females) were collected from Lanping black-boned sheep (LPBB) as the experimental group in Lanping County along with 50 blood samples (of 25 males and 25 females) from Lanping normal sheep (LPN) and another 50 blood samples (of 25 males and 25 females) from Huize normal sheep (HZN) as the control group in Huize County, Yunnan Province, China. Each individual was over 1 year old; carefully examined according to traits and parentage to ensure adherence to breed standards; marked with sampling site, species, sex, and age group; and transported to the laboratory as soon as possible. The care and use of animals fully complied with local animal welfare laws, guidelines, and policies. Subsequently, a portion of each blood sample was centrifuged to determine melanin indexes, while the remaining samples were stored at - 20 °C for genotype analysis.

### 2.3 DNA extraction and PCR amplification

Sheep genomic DNA was extracted from whole blood using standard proteinase K digestion followed by extraction with phenol–chloroform as described by Sambrook et al. (2006) (Yang et al., 2021; Bo et al., 2021). Based on public sequences of the *PDGFRL* gene of sheep (sequence number NC_040277) and six exon regions, six exon primers were designed using the Premier 6.0 software which were subsequently subjected to primer specific detection in the NCBI online database (https://www.ncbi.nlm.nih.gov/tools/primer-blast/, last access: 15 June 2023). After the design was completed, it was sent to Kunming Shuoqing Biotechnology Co., Ltd. to synthesize PCR primers. The six pairs of primer sequences of *PDGFRL* gene are shown in Table 1.

The PCR reactions were executed in a total of 25.0 µ L volume, which contained 1 µ L of genomic DNA (20 ng µ L ${}^{-1}$ ); 22 µ L of mix (green) from Beijing Qingke Biotechnology Co., Ltd. (https://www.chem960.com/offer/bio1398078/, last access: 21 June 2023); and 1 µ L of upper and lower primers (10 µ mol µ L ${}^{-1}$ ). After many explorations, PCR amplification was performed using the following programs. EX1–EX3 and EX5 reaction conditions consisted of the initial denaturing at 98 °C for 2 min followed by 30 cycles of denaturing at 98 °C for 10 s, 10 s of annealing, and 10 s of elongating at 72°C, with a final extension at 72°C for 1 min; EX4 and EX6 reaction conditions consisted of the initial denaturing at 94 °C for 4 min followed by 30 cycles of denaturing at 94 °C for 30 s, with the annealing temperature shown in Table 1.

**Table 1:** Information on the primer sequence.

Primer	Primer	Product	Annealing	Amplification
	sequence	size	temperature	location
	( $5^{\prime }\to 3^{\prime }$ )	[bp]	[°C]	
*PDGFRL*-EX1	F: GGCAGGAGAGGTCACATTACACA R: GGGAGTGGAGAGAGGGAGGTAAG	449	61.0	Partial 5 ${}^{\prime }$ -UTR, complete exon 1,partial intron 1
*PDGFRL*-EX2	F: CCTTGGAGGTGGTGAGACTGAG R: AGCAACACGACTAGTGAAGACCAA	502	58.5	Partial intron 1, complete exon 2,partial intron 2
*PDGFRL*-EX3	F: CCTCCTCACCTACCCCAACAC R: CACTTCTTGCCATCTGACCTGA	592	58.5	Partial intron 2, complete exon 3,partial intron 3
*PDGFRL*-EX4	F: TCGCATCTTTGTGCCCTGAA R: AGCCATCTGTGCCTCCTACT	484	60.0	Partial intron 3, complete exon 4,partial intron 4
*PDGFRL*-EX5	F: AGAGGCATCATAAAACCCCAAG R: ATTTTAGCAGCAAACCAGCAAGT	462	55.0	Partial intron 4, complete exon 5,partial intron 5
*PDGFRL*-EX6	F: TTATGCCTAATACCAGCCCCAC R: GTCTGGGTCTGACTCACAAGGC	796	59.0	Partial intron 5, complete exon 6,partial 3 ${}^{\prime }$ -UTR

### 2.4 Determination of melanin indexes in plasma

The activity of tyrosinase (TYR) was measured using a UV–visible spectrophotometer (HP8453, Germany) at 475 nm, with L-DOPA serving as the substrate (Pomerantz, 1963). Plasma colorimetric absorbance was evaluated at 475 nm using distilled water as a blank control after combining the plasma with physiological saline in a 1:7 ratio (Nam et al., 2021). The preparation of TYR solutions with different concentrations is shown in Table 2.

**Table 2:** The configuration of different concentrations of the TYR standard solution.

Concentration of TYR	Amount of TYR	Amount of
standard solution	standard solution	0.1 mol L ${}^{-1}$ (pH = 7.0)
[mg mL ${}^{-1}$ ]	at 0.5 mg mL ${}^{-1}$ [ µ L]	phosphate buffer [ µ L]
0.0625	62.5	437.5
0.0925	92.5	407.5
0.1250	125	375
0.1550	155	345
0.2500	250	250

Phaeomelanin content was determined by separating 800 µ L 0.1 M PBS (pH = 10.5), adding a 200 µ L plasma sample and 1 mL chloroform to the Eppendorf centrifuge tube, vortex shaking and mixing well, and then centrifuging at 10 000 rpm for 10 min. Subsequently, the supernatant was taken and the absorbance at a wavelength of 400 nm measured using a 722 spectrophotometer (the blank control is phosphate buffer with pH = 10.5).

Alkaline-soluble melanin content was determined by separating 800 µ L urea(8 M) / NaOH(1 M), adding a 200 µ L plasma sample and 1 mL chloroform to the Eppendorf centrifuge tube, vortex shaking and mixing well, and then centrifuging at 10 000 rpm for 10 min. Subsequently, the supernatant was taken and the absorbance at a wavelength of 400 nm measured using a 722 spectrophotometer (the blank control is urea(8 M) / NaOH(1 M).).

Total melanin content in plasma was determined by adding 900 µ L Soluene-350 to the test tube and a 100 µ L mix of different plasma samples by suction and beating and then heating the mix in boiling water for 45 min, cooling it to room temperature, and centrifuging it at 10 000 rpm for 10 min. Subsequently, the upper layer of brownish yellow clear liquid is taken and the absorbance at a wavelength of 500 nm measured using a 722 spectrophotometer (the blank control is Soluene-350).

Plasma true melanin / total melanin was measured by adding 900 µ L Soluene-350 and a 100 µ L plasma sample to the test tube, aspirating and mixing the resulting mix evenly, heating it in boiling water for 45 min, and then cooling and centrifuging it at 10 000 rpm for 10 min. Subsequently, the upper layer of brownish yellow clear liquid is taken and the absorbance values (A500 and A650) at wavelengths of 500 and 650 nm measured using a 722 spectrophotometer. The blank control is Soluene-350, and the A500 / A650 values are calculated. Plasma colorimetry is determined by mixing plasma with physiological saline at a ratio of 1:7 and then measuring the absorbance value at a wavelength of 475 nm using a 722 spectrophotometer (blank control was physiological saline).

### 2.5 Sequencing and sequence analysis

The PCR products were excised form 1 % agarose gel and then sent to Beijing Tsingke Biotechnology Co., Ltd. for direct sequencing. The sequencing results were first proofread with a DNA peak form graph using Chromas v.2.6 (Davide et al., 2016) and compared using BLASTN (http://www.ncbi.nlm.nih.gov, last access: 11 July 2023). Ultimately, the checked sequences were amended by aligning with reference sequences downloaded from the GenBank database using the software Clustal X v.1.83 (Thompson et al., 1997) to identify whether all three mutation sites (EX2-G408A, EX5-T184C, and EX5-G222T) were successfully amplified. The nomenclature of novel sequences was identified in this study following that of previous studies (James et al., 2017; Zhong et al., 2017).

### 2.6 Statistical analysis

The differences in sheep between different breeds, sexes, age groups, and locations were calculated using a chi-square test in SPSS 25.0 (SPSS Inc., Chicago, IL, USA). All data with a value of P<0.05 were measured to be statistically significant.

## 3 Results

### 3.1 Analysis of sheep melanin indexes in plasma

In the course of this investigation, a comprehensive evaluation encompassing 150 sheep facilitated the measurement of melanin indexes in plasma. Strikingly, with the exception of total melanin, all other pertinent melanin metrics exhibited noteworthy disparities, unequivocally underscoring the distinctive melanin profile of LPBB in comparison to LPN. Notable elevations were observed in TYR activity (446.858 ± 11.933 IU mL ${}^{-1}$ ), eumelanin / total melanin ratio (0.395 ± 0.013), phaeomelanin, alkaline-soluble melanin, and plasma colorimetry (0.181 ± 0.010 OD mL ${}^{-1}$ ) within the LPBB cohort, all of which achieved statistical significance ( P<0.05 ). Furthermore, variables such as group, gender, and age exhibited a discernible influence on each melanin index, with statistical significance attained as well ( P<0.05 ). The intricate interplay between LPBB and LPN was meticulously explored, revealing distinct variations in melanin content, as elucidated in Table 3. This nuanced analysis not only unveils the inherent differences in melanin profiles between the two sheep populations but also underscores the multifactorial nature of melanin regulation, with potential implications for understanding the broader spectrum of genetic and environmental influences on melanogenesis in ovine species.

**Table 3:** Comparison of melanin content between LPBB and normal sheep and variance analysis of related factors.

Indicator	LPBB			LPN			Influencing factor					
	Min	Max	Mean + SD	Min	Max	Mean + SD	Group	Gender	Age	Group + gender	Group + age	Gender + age
TYR activity	291.577	562.480	446.858 ± 11.933 ${}^{a}$	289.827	336.821	322.484 ± 11.407 ${}^{b}$	4.21 × 10 ${}^{-9^{*}}$	0.105	0.045 ${}^{*}$	1.40 × 10 ${}^{-9^{*}}$	2.10 × 10 ${}^{-9^{*}}$	0.259
[IU mL ${}^{-1}$ ]												
Phaeomelanin	0.167	0.282	0.230 ± 0.006 ${}^{a}$	0.116	0.206	0.158 ± 0.008 ${}^{b}$	0.0002 ${}^{*}$	0.167	0.149	0.0005 ${}^{*}$	0.005 ${}^{*}$	0.216
[OD mL ${}^{-1}$ ]												
Alkaline-soluble melanin	0.157	0.236	0.200 ± 0.010 ${}^{a}$	0.078	0.148	0.110 ± 0.004 ${}^{b}$	1.61 × 10 ${}^{-8^{*}}$	0.080	0.289	0.169	0.021 ${}^{*}$	0.011 ${}^{*}$
[OD mL ${}^{-1}$ ]												
Total melanin	0.134	0.485	0.333 ± 0.012	0.109	0.384	0.309 ± 0.009	0.151	0.782	0.490	0.094	0.100	0.543
[OD mL ${}^{-1}$ ]												
Eumelanin /	0.278	0.452	0.395 ± 0.013 ${}^{a}$	0.217	0.297	0.224 ± 0.005 ${}^{b}$	6.10 × 10 ${}^{-8^{*}}$	0.008 ${}^{*}$	0.020 ${}^{*}$	0.010 ${}^{*}$	0.042 ${}^{*}$	0.020 ${}^{*}$
total melanin												
Plasma colorimetry	0.095	0.207	0.181 ± 0.010 ${}^{a}$	0.058	0.097	0.077 ± 0.008 ${}^{b}$	0.50 × 10 ${}^{-5^{*}}$	0.447	0.958	0.300	0.105	0.535
[OD mL ${}^{-1}$ ]												

Note that for the comparison between two sheep groups, different letters, A and B, indicate a significant difference ( P<0.05 ) for LPBB and normal sheep, respectively, while no annotation indicates no significant difference ( P>0.05 ). In variance analysis of index influencing factors, values marked with ${}^{*}$ indicate a significant influence ( P<0.05 ), while those unmarked indicate that there is no significant difference ( P>0.05 ).

### 3.2 PCR amplification

**Figure 1:**
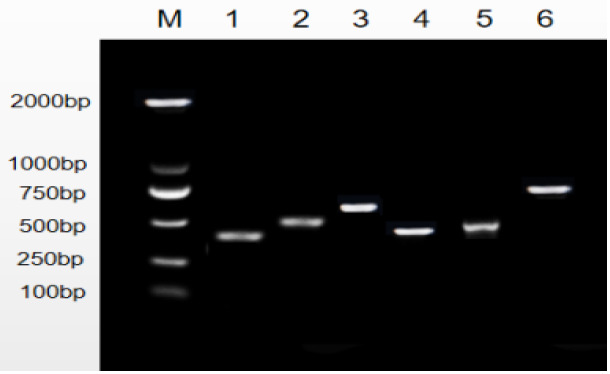
Electrophoresis map of *PDGFRL* gene exon amplification in LPBB. Note that M represents the size of the marker band and numbers 1-6 represent the size of the bands amplified by six primers, respectively.

**Figure 2:**
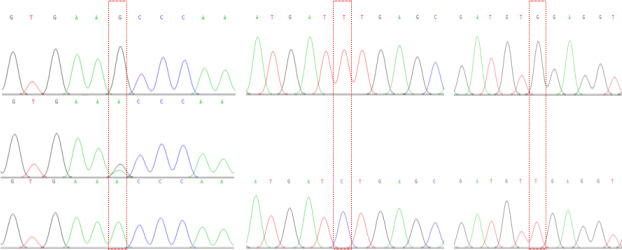
Mutation sites (G408A, T184C, and G222T).

The amplification of PCR fragments using the six primer pairs yielded distinct sizes, measuring 449, 502, 592, 484, 462, and 796 bp, respectively, as illustrated in Fig. 1. Subsequent scrutiny revealed three discernible polymorphic sites, EX2-G408A, EX5-T184C, and EX5-G222T, depicted in Fig. 2. Remarkably, the complete coding regions encompassing 375 amino acids were successfully delineated from LPBB, LPN, and HZN. What is noteworthy is the absence of any non-synonymous mutations, affirming the preservation of the amino acid sequence. Intriguingly, an expansive comparative analysis illuminated variations in the *PDGFRL* gene across diverse ruminant species. The highest sequence identity was noted in goats, at an impressive 98.76 %, succeeded by cattle (96.99 %), deer (95.74 %), and horses (88.22 %). This observed diversity in sequence homology underscores the nuanced evolutionary dynamics shaping the *PDGFRL* gene within the broader context of ovine genetics, offering valuable insights into the intricate interplay of genetic conservation and divergence across distinct ruminant lineages.

**Figure 3:**
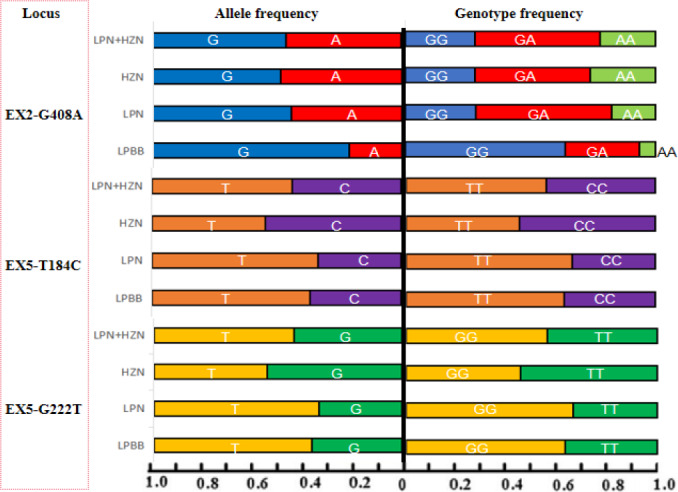
The genotype and gene frequencies of polymorphic sites of the *PDGFRL* gene in sheep.

### 3.3 Allele and genotype frequencies of the *PDGFRL* gene

Figure 3 elucidates the genotypic landscape and allele frequencies within the *PDGFRL* gene across the three distinct sheep groups under scrutiny. LPBB exhibited a notable preference for GG and GA genotypes, juxtaposed against LPN and HZN, wherein the GA genotype dominated at the EX2-G408A site. In LPBB, the GG and GA genotypes were notably favored in contrast to LPN and HZN, where the GA genotype predominated at the EX2-G408A site. The allelic distribution showcased a frequency of 0.788 for allele G in LPBB, while LPN and HZN exhibited frequencies of 0.554 and 0.510, respectively. Upon subjecting the populations to the rigors of the Hardy–Weinberg equilibrium through chi-square testing, incongruities emerged. LPBB and LPN manifested an augmented frequency of TT and a diminished frequency of CC at the EX5-T184C site, mirroring a parallel trend at the EX5-G222T site. Intriguingly, HZN exhibited a converse pattern with an elevated frequency of CC and a reduced frequency of TT at both loci. These observed genotype distributions yielded statistically significant differences ( P<0.01 ), signaling a departure from the expected equilibrium state. This departure from the Hardy–Weinberg equilibrium underscores the intricate dynamics governing the genetic interplay within each sheep population, inviting further exploration into the underlying genetic forces shaping *PDGFRL* gene polymorphism in these distinct ovine cohorts.

### 3.4 Correlation analysis of *PDGFRL* gene polymorphism and melanin traits

Within the confines of this investigation, LPBB bearing GG and GA genotypes distinctly exhibited a statistically significant elevation ( P<0.05 ) in TYR activity at the EX2-G408A site, as delineated in Table 3. Furthermore, TT and GG genotypes demonstrated a significant association ( P<0.05 ) with augmented eumelanin / total melanin ratios at the EX5-T148C and EX5-G222T sites, respectively. Intriguingly, LPBB individuals harboring GG, TT, and GG genotypes across these three sites showcased a remarkable and statistically significant increase ( P<0.05 ) in TYR activity, eumelanin / total melanin ratios, and plasma colorimetry when juxtaposed against their normal sheep counterparts. This discernment accentuates the pivotal role of genotypic variations at these specific loci in orchestrating the heightened melanogenic attributes observed in LPBB, unraveling a nuanced interplay between the *PDGFRL* gene polymorphism and melanin-related phenotypic traits.

**Table 4:** Association analysis of polymorphism of *PDGFRL* gene and melanin traits in LPBB.

Polymorphic site	Index	Sheep type					
		LPBB			Normal sheep		
		GG	GA	AA	GG	GA	AA
EX2-G408A	TYR activity	477.117 ± 23.995 ${}^{\mathrm{Aa}}$	414.822 ± 21.735 ${}^{a}$	272.145 ± 11.746 ${}^{b}$	333.841 ± 13.151 ${}^{B}$	319.422 ± 19.787	319.014 ± 18.943
	Eumelanin / total melanin	0.316 ± 0.011 ${}^{A}$	0.281 ± 0.012 ${}^{A}$	0.296 ± 0.023 ${}^{A}$	0.245 ± 0.009 ${}^{B}$	0.243 ± 0.008 ${}^{B}$	0.250 ± 0.008 ${}^{B}$
	Total melanin	0.333 ± 0.017	0.373 ± 0.020	0.336 ± 0.028	0.369 ± 0.019	0.337 ± 0.015	0.323 ± 0.014
	Phaeomelanin	0.258 ± 0.019 ${}^{\mathrm{Aa}}$	0.269 ± 0.021 ${}^{\mathrm{Aa}}$	0.185 ± 0.025 ${}^{b}$	0.169 ± 0.011 ${}^{B}$	0.167 ± 0.014 ${}^{B}$	0.161 ± 0.012
	Alkaline-soluble melanin	0.175 ± 0.010 ${}^{A}$	0.188 ± 0.011 ${}^{A}$	0.157 ± 0.025 ${}^{A}$	0.106 ± 0.009 ${}^{B}$	0.095 ± 0.008 ${}^{B}$	0.094 ± 0.007 ${}^{B}$
	Plasma colorimetry	0.155 ± 0.015 ${}^{A}$	0.125 ± 0.014 ${}^{A}$	0.129 ± 0.016 ${}^{A}$	0.080 ± 0.015 ${}^{B}$	0.072 ± 0.009 ${}^{B}$	0.064 ± 0.007 ${}^{B}$
		TT	TC	CC	TT	TC	CC
EX5-T184C	TYR activity	426.793 ± 27.621 ${}^{A}$	NC	371.287 ± 37.198	331.130 ± 18.678 ${}^{B}$	NC	336.530 ± 10.683
	Eumelanin / total melanin	0.346 ± 0.022 ${}^{\mathrm{Aa}}$	NC	0.267 ± 0.005 ${}^{b}$	0.244 ± 0.013 ${}^{B}$	NC	0.239 ± 0.012
	Total melanin	0.319 ± 0.021	NC	0.362 ± 0.019	0.355 ± 0.018	NC	0.344 ± 0.017
	Phaeomelanin	0.223 ± 0.012	NC	0.210 ± 0.013 ${}^{A}$	0.204 ± 0.012 ${}^{a}$	NC	0.164 ± 0.010 ${}^{\mathrm{Bb}}$
	Alkaline-soluble melanin	0.170 ± 0.014	NC	0.159 ± 0.010	0.172 ± 0.015	NC	0.136 ± 0.013
	Plasma colorimetry	0.130 ± 0.019 ${}^{A}$	NC	0.108 ± 0.013 ${}^{A}$	0.081 ± 0.042 ${}^{B}$	NC	0.063 ± 0.004 ${}^{B}$
		GG	GT	TT	GG	GT	TT
EX5-G222T	TYR activity	426.793 ± 27.621 ${}^{A}$	NC	371.287 ± 37.198	331.130 ± 18.678 ${}^{B}$	NC	336.530 ± 10.683
	Eumelanin / total melanin	0.346 ± 0.022 ${}^{\mathrm{Aa}}$	NC	0.267 ± 0.005 ${}^{b}$	0.244 ± 0.013 ${}^{B}$	NC	0.239 ± 0.012
	Total melanin	0.319 ± 0.021	NC	0.362 ± 0.019	0.355 ± 0.018	NC	0.344 ± 0.017
	Phaeomelanin	0.223 ± 0.012	NC	0.210 ± 0.013 ${}^{A}$	0.204 ± 0.012 ${}^{a}$	NC	0.164 ± 0.010 ${}^{\mathrm{Bb}}$
	Alkaline-soluble melanin	0.170 ± 0.014	NC	0.159 ± 0.010	0.172 ± 0.015	NC	0.136 ± 0.013
	Plasma colorimetry	0.130 ± 0.019 ${}^{A}$	NC	0.108 ± 0.013 ${}^{A}$	0.081 ± 0.042 ${}^{B}$	NC	0.063 ± 0.004 ${}^{B}$

Note that capital letters represent the same genotype, with significant differences between different sheep categories; lowercase letters represent the same sheep category with significant differences between different genotypes; and NC represents the fact that the genotype has not been detected.

## 4 Discussion

In this investigation, we pioneered the acquisition of the complete *PDGFRL* gene sequence from LPBB through comparative genomics. While the *PDGFRL* gene has predominantly garnered attention in human studies (Liu et al., 2022; Meena et al., 2012) as well as in investigations of chickens (McDerment et al., 2015) and mice (Kawata et al., 2017), prior research has hinted at its regulatory role in the growth and development of layer follicles (Yang et al., 2016). Furthermore, evidence implicates the *PDGFRL* gene in tumor progression and metastasis, particularly in melanoma (Xu et al., 2020b; Guo et al., 2010; Wenbin et al., 2008). Surprisingly, the potential impact of the *PDGFRL* gene on melanin synthesis in LPBB or any other ruminant has hitherto remained veiled. Compellingly, indirect interactions with key signaling proteins, including *MITF*, CREB, β -catenin, transforming growth factor beta (TGF- β ), and proton-activated chloride (PAC) channel, in the regulatory cascade of melanin synthesis have been suggested in the literature (Gelmi et al., 2022; Chowdhury et al., 2023; Liu et al., 2022b; Peng et al., 2022; Rodriguez et al., 2018).

Therefore, the comprehensive analysis of DNA polymorphisms within three melanin trait genes, which distinctively deviate from the melanocyte distribution in LPBB and local normal sheep, provides a theoretical foundation for unraveling the intricacies of melanin transfer mechanisms. Extensive research has demonstrated that the heightened eumelanin content constitutes the primary determinant of LPBB's distinctive melanin characteristics, which are contingent on elevated TYR activity levels (Song et al., 2023; Koehler et al., 2021). Notably, elevated TYR activity tends to favor increased eumelanin production, while diminished TYR activity is conducive to phaeomelanin production (Guo et al., 2023; Shinomiya et al., 2012).

In the context of this experiment, LPBB exhibited significantly higher levels ( P<0.05 ) of TYR activity (446.858 ± 11.933 IU mL ${}^{-1}$ ), eumelanin / total melanin ratio (0.395 ± 0.013), phaeomelanin, alkaline-soluble melanin, and plasma colorimetry (0.181 ± 0.010 OD mL ${}^{-1}$ ) in comparison to LPN, and these findings also cohesively align with prior research attributing diverse edible properties to LPBB in contrast to LPN (Sun et al., 2020; Chen et al., 2019). This confluence of evidence reinforces the intricate link between the *PDGFRL* gene, the melanin synthesis, and the distinctive phenotypic traits observed in LPBB, offering novel insights into the molecular underpinnings of melanin regulation in ovine species.

Furthermore, the amplification of the complete coding region of the *PDGFRL* gene unearthed three silent mutation sites at EX2-G408A, EX5-T184C, and EX5-G222T. Within the mixed sheep group, individuals bearing GG and GA genotypes exhibited a pronounced elevation ( P<0.05 ) in TYR activity at the EX2-G408A site, while those with TT and GG genotypes displayed a significant increase ( P<0.05 ) in eumelanin / total melanin ratios at the EX5-T184C and EX5-G222T sites, respectively. Despite the synonymous nature of these mutations, which implies an unaltered amino acid sequence, their potential impact on mRNA levels was postulated, implicating disturbances in the exon splicing enhancer gene sequence within the corresponding region. This disruption could potentially lead to truncated proteins, as suggested by the existing literature (Shen et al., 2022; Wang et al., 2023; Liu et al., 2020). Additionally, the influence of synonymous mutations on the three-dimensional structure and functional domains of proteins has been documented in the scientific discourse (Li et al., 2021).

Our results indicate the presence of synonymous mutations in the *PDGFRL* gene, and speculation that these synonymous mutations may affect the function of the *PDGFRL* gene by altering its conformation, which in turn affects the inhibitory effects on melanocyte differentiation, division, and metastasis, requires further supporting evidence.

## 5 Conclusions

This study investigated melanin indexes in sheep blood and found that Lanping black-boned sheep (LPBB) exhibited significantly higher melanin indexes ( P<0.05 ) than normal sheep. Three synonymous mutation sites were identified at EX2-G408A, EX5-T184C, and EX5-G222T in a partial segment of 1128 bp of the exon of the gene-encoding *PDGFRL*. Moreover, there was a significant difference ( P<0.05 ) in gene distribution between the mixed population of sheep, and the GG, TT, and GG genotypes at the three sites were identified as the dominant genotypes of LPBB, respectively. However, the gene was not missense mutated, and further validation is needed to determine whether it affects changes in melanin content.

## Data Availability

The data that support the findings of this study are available upon request from the corresponding author via email.
